# Clinical course of Coronavirus Disease-19 in patients with haematological malignancies is characterized by a longer time to respiratory deterioration compared to non-haematological ones: results from a case–control study

**DOI:** 10.1007/s15010-022-01869-w

**Published:** 2022-07-03

**Authors:** A. Oliva, A. Curtolo, L. Volpicelli, F. Cancelli, C. Borrazzo, F. Cogliati Dezza, G. Marcelli, F. Gavaruzzi, S. Di Bari, P. Ricci, O. Turriziani, C. M. Mastroianni, M. Venditti

**Affiliations:** 1 Department of Public Health and Infectious Diseases, Sapienza University of Rome, Piazzale Aldo Moro, 500185 Rome, Italy; 2 Department of Medico-Surgical Sciences and Biotechnologies, Sapienza University of Rome, Rome, Italy; 3 Unit of Emergency Radiology, Department of Radiological, Oncological and Pathological Sciences, Sapienza University of Rome, Rome, Italy; 4 Microbiology and Virology Laboratory, Department of Molecular Medicine, Sapienza University of Rome, Rome, Italy

**Keywords:** Haematological malignancy, SARS-CoV2, COVID-19, Multiple myeloma, Non-Hodgkin lymphoma

## Abstract

**Background:**

We evaluated clinical features and risk factors for mortality in patients with haematological malignancies and COVID-19.

**Methods:**

Retrospective, case–control (1:3) study in hospitalized patients with COVID-19. Cases were patients with haematological malignancies and COVID-19, controls had COVID-19 without haematological malignancies. Patients were matched for sex, age and time of hospitalization.

**Results:**

Overall, 66 cases and 198 controls were included in the study. Cases had higher prior corticosteroid use, infection rates, thrombocytopenia and neutropenia and more likely received corticosteroids and antibiotics than controls. Cases had higher respiratory deterioration than controls (78.7% vs 65.5%, *p* = 0.04). Notably, 29% of cases developed respiratory worsening > 10 days after hospital admission, compared to only 5% in controls. Intensive Care Unit admission and mortality were higher in cases than in controls (27% vs 8%, *p* = 0.002, and 35% vs 10%, *p* < 0.001).

At multivariable analysis, having haematological malignancy [OR4.76, *p* < 0.001], chronic corticosteroid therapy [OR3.65, *p* = 0.004], prior infections [OR57.7, *p* = 0.006], thrombocytopenia [OR3.03, *p* < 0.001] and neutropenia [OR31.1, *p* = 0.001], low albumin levels [OR3.1, *p* = 0.001] and ≥ 10 days from hospital admission to respiratory worsening [OR3.3, *p* = 0.002] were independently associated with mortality.

In cases, neutropenia [OR3.1, *p* < 0.001], prior infections [OR7.7, *p* < 0.001], ≥ 10 days to respiratory worsening [OR4.1, *p* < 0.001], multiple myeloma [OR1.5, *p* = 0.044], the variation of the CT lung score during hospitalization [OR2.6, *p* = 0.006] and active treatment [OR 4.4, *p* < 0.001] all were associated with a worse outcome.

**Conclusion:**

An underlying haematological malignancy was associated with a worse clinical outcome in COVID-19 patients. A prolonged clinical monitoring is needed, since respiratory worsening may occur later during hospitalization.

**Supplementary Information:**

The online version contains supplementary material available at 10.1007/s15010-022-01869-w.

## Introduction

QuerySevere acute respiratory syndrome coronavirus-2 (SARS-CoV2) was first reported in late December 2019 and was recognized as the causative agent of an acute respiratory tract infection named Coronavirus Disease 2019 (COVID-19) [1]. Although most patients were asymptomatic or exhibited mild respiratory symptoms, approximately 8% may require hospitalization and 1% admission in Intensive Care Unit (ICU) [2]. In this context, patients suffering from haematological malignancies were considered an extremely vulnerable group, with a high risk of severe illness [3, 4] due to the prolonged and severe immunosuppression resulting from both chemo-immunotherapy and the underlying disease, which may weaken the immune system [5]. Indeed, the severe immunosuppression and the potential delay in the underlying disease’s treatment due to SARS-CoV2 diagnosis may affect the outcome of patients with haematological malignancies and COVID-19. On the other hand, these patients might also be protected from severe forms of COVID-19 thanks to the lower immune reaction during SARS-CoV2 infection [6, 7].

Although the relation between haematological malignancies and severity of SARS-CoV2 disease has been widely demonstrated, still no clear data on the risk factors of mortality in this special population exist. Furthermore, it is not fully elucidated whether there are differences in the clinical course of COVID-19 disease between patients with or without haematological malignancies.

As a matter of fact, so far the majority of studies have included mixed cohorts combining patients with haematological and solid cancer [3, 8–10]. Whereas, case–control studies, specifically matching subjects with and without haematological malignancies affected by SARS-CoV2, are still rare and include only a few patients [11, 12].

Based on these premises, the primary aim of this case–control study was to evaluate the clinical and prognostic features of patients with haematological malignancies and COVID-19. The secondary aim was to analyse risk factors for in-hospital mortality in this special population, with a particular emphasis on the respiratory worsening during hospitalization.

## Materials and methods

From April 2020 to April 2021, a retrospective, single-center, case–control (1:3) study was performed on patients with COVID-19 hospitalized at Azienda Ospedaliero-Universitaria Policlinico Umberto I, Sapienza University of Rome. Cases were patients with haematological malignancies and COVID-19. Controls were patients with COVID-19 but without haematological malignancies. Cases and controls were matched for sex, age (decades) and time of hospitalization (Supplementary Fig. 1).

Inclusion criteria were: i) diagnosis of COVID-19 by means of molecular analyses, ii) hospitalization, and iii) age > 18 years. Patients with haematological disorders other than malignancy and age < 18 years were excluded from the study.

Nasopharyngeal swab samples were collected and SARS-CoV-2 RNA was detected using real-time RT-PCR assay (RealStar SARS-CoV2 RT-PCR, Altona Diagnostics).

For each subject, laboratory and clinical data at hospital admission, as well as treatment data and SARS-CoV-2 RNA time of detection, were collected and recorded anonymously in an electronic database. As for treatment data, antiviral therapy and the choice of using steroids were based on the national, regional and local guidelines available at the time [13, 14] and on clinical judgement, also following consultation with haematologists when appropriate.

In patients with haematological malignancies, the following variables were also collected: type and status of underlying disease, rate and type of active treatment, prior (30 d) chemotherapy, percentage of total lung parenchyma involvement during the disease by means of CT scan, CT score.

### Definitions

Severity of infection was defined according to the guidelines available at the time of enrolment [15]. Individuals who tested positive for SARS-CoV2 but had no related symptoms were considered asymptomatic [15].

Use of corticosteroids within the previous 30 days included therapy with prednisone or its equivalent at a dose > 0.5 mg/kg/day for at least 1 month.

Prior infection and antibiotic therapy were defined as a diagnosis of infection and/or the receival of antibiotics in the 30 d prior to hospital admission, respectively.

Thrombocytopenia and neutropenia were defined according to the guidelines [16].

Status of haematological malignancy was defined as new diagnosis, refractory or relapsing disease, according to the guidelines of European Society for Medical Oncology [17].

Prior active treatment included the receival of chemotherapy or immunotherapy, or both, in the previous 90 days. Immunotherapy included the receival of monoclonal antibodies (rituximab, daratumumab, obinutuzumab) and tyrosine kinase inhibitors (imitinib, ibrutinib, ruxolitinib, venetoclax).

Worsening of respiratory conditions was based on the change of PaO2/FiO2 and was defined as: i) the need of supplementary oxygen therapy or ii) the need of increasing oxygen therapy supplementation in a patient with SARS-CoV2 infection for reasons directly related to the infection. A careful evaluation of causes of supplementary oxygen therapy for reasons other than SARS-CoV2 infection (i.e. cardiac failure, bacterial superinfections) was performed. In the case of doubt, a panel discussion was performed.

Time of viral shedding was defined as the number of days from the first viral detection by RT-PCR on nasopharyngeal specimen until the first of two consecutive negative results within 24 h [18].

To understand the dynamic of radiological findings in patients with haematological malignancies, we performed a CT scan at the diagnosis (CT1) and at the time of respiratory worsening (CT2). Two multidetector CT scanners (Somatom Sensation 16 and Somatom Sensation 64; Siemens Healthineers) were used. COVID-19 pneumonia was confirmed by means of the following CT parameters: ground glass opacity, crazy-paving pattern and pulmonary consolidation [19]. A semi-quantitative CT severity score to identify the percentage of lung parenchyma involved by the infective process was therefore calculated. In detail, each lung was divided into upper, middle and lower zones. For each of 3 zone, the extent of anatomic involvement was calculated as follows: 0, no involvement; 1 < 25% involvement; 2, 25–50% involvement; 3, 51–75% involvement; 4 > 75% involvement. The resulting global CT score was the sum of each individual score, ranging from 0 to 24 [20].

The study was approved by the local Ethics Committee (ID Prot. 109/2020).

### Statistical analyses

Continuous data were expressed as mean with Standard Deviation (± SD) and categorical data were summarized as number of observations (n) and percentages (%). Differences between groups were evaluated with unpaired *T* test; difference between proportions was calculated by Chi-square test. Binary logistic regression analysis was used to identify the demographic characteristics and the examined risk factors. Factors with a univariate value of *p* < 0.05 were included in a stepwise multivariate logistic regression analysis. Missing data for each variable were excluded from the denominator. Univariable analysis was used to identify risk factors for haematological or non-haematological and predictors of all cause 30 day mortality. Baseline predictors possibly associated with the outcome at univariable comparison were considered for multivariable logistic regression to estimate adjusted Odds Ratios (ORs) and 95% Confidence Intervals (95% CI) for the risk factors including the confounding factors (i.e., age, gender). A stepwise backward selection approach was used to select the predictors to include in the final multivariate model. Survival was analysed by Kaplan–Meier curves and the statistical significance of the differences between the two groups was assessed using the log-rank test. Statistical analyses were performed using statistical Program for the Social Sciences (SPSS, version 22, SPSS Inc. Chicago IL, USA) software package.

## Results

A total of 264 patients were included in the study (66 cases and 198 controls) (Supplementary Fig. 1). General features of the study population are shown in Table 1. Age, gender distribution and period of hospitalization did not differ between cases and controls, reflecting the correctness of the match. Likewise, infection severity and the PaO2/FiO2 at hospital admission were similar between cases and controls [(asymptomatic 4% vs 6%, *p* = 0.438, moderate infection 64% vs 64%, *p* = 0.904, severe infection 27% vs 29%, *p* = 0.796, critical illness 5% vs 6%, *p* = 0.619, respectively), 357.0 ± 102.1 vs 352.4 ± 101.0, *p* = 0.750]. Compared to controls, cases had a higher rate of chronic corticosteroids use and infections in the previous 30 d (39% vs 10%, *p* < 0.001 and 21% vs 3%, *p* = 0.001, respectively), as well as higher percentage of thrombocytopenia (39% vs 18%, *p* = 0.002), neutropenia (14% vs 1%, *p* = 0.003) and lower albumin levels (35.6 ± 6.7 vs 37.4 ± 5.5 g/dL, *p* = 0.003). C-reactive protein values did not differ between cases and controls (6.57 ± 8.41 vs 7.06 ± 7.52 mg/dL, *p* = 0.65).

**Table 1:** General features of study population

	Cases (*N* = 66)	Controls (*N* = 198)	*p* value
Demographics			
Gender (Female), *n* (%)	32 (48)	96 (48)	1.000
Age, mean (SD)	62.14 (± 15.37)	61.28 (± 14.4)	0.697
Steroids in the last 30 days before admission, *n* (%)	26 (39)	19 (10)	< 0.001
Infection in the last 30 days before admission, *n* (%)	14 (21)	5 (3)	0.001
Antibiotic therapy in the last 30 days (excluded prophylaxis), *n* (%)	22 (33)	69 (35)	0.883
Antibiotic prophylaxis therapy, *n* (%)	24 (36)	0 (0)	< 0.001
Days of hospitalization, mean (SD)	31.79 (± 28.04)	19.16 (± 13.4)	0.001
Days from symptoms onset to hospitalization, mean (SD)	5.48 (± 9.03)	6.72 (± 6.02)	0.310
Complete vaccination for SARS-CoV-2, *n* (%)	1 (1.5)	1 (0.5)	0.9
Comorbidities, *n* (%)			
Hypertension	23 (35)	93 (47)	0.082
Coronary artery disease	14 (21)	18 (9)	0.029
Diabetes	9 (14)	33 (17)	0.547
Chronic obstructive pulmonary disease	4 (6)	17 (9)	0.481
Chronic renal failure	6 (9)	10 (5)	0.302
Disease severity at the admission, *n* (%)			
Moderate	42 (64)	127 (64)	0.904
Severe	18 (27)	57 (29)	0.796
Critical	3 (5)	12 (6)	0.619
Pneumonia	55 (83)	143 (72)	0.169
Symptoms at the admission, *n* (%)			
Dyspnea	26 (39)	116 (59)	0.007
Cough	24 (36)	100 (51)	0.044
Asthenia	28 (42)	54 (27)	0.197
Anosmia/ageusia	4 (6)	24 (12)	0.109
Fever	46 (70)	159 (80)	0.099
Conjunctivitis	2 (3)	12 (6)	0.267
Other*	1 (2)	0 (0)	0.322
Asymptomatic	4 (6)	7 (4)	0.438
Respiratory features at the admission, mean (SD)			
SpO_2_	95.6 (± 5.2)	95.3 (± 3.2)	0.680
PO_2/_FiO_2_	357.0 (± 102.1)	352.4 (± 101)	0.750
FiO_2_	24.6 (± 8.5)	24.2 (± 9.3)	0.737
Laboratory findings at the admission, mean (SD)			
Haemoglobin, g/dl	11.4 (± 2.0)	13.4 (± 2.1)	< 0.001
White Blood Cells, × 10˄6/L	8761.4 (± 10,816.8)	7762.7 (± 9170.8)	0.502
Neutrophils, × 10˄6/L	5697.8 (± 7252.7)	5610.8 (± 3740.4)	0.926
Lymphocytes, × 10˄6/L	2209.4 (± 6128.7)	1043.0 (± 522.5)	0.127
Monocytes, × 10˄6/L	345.5 (± 385.8)	369.2 (± 194.56)	0.633
Platelets, × 10˄9/L	172.6 (± 114.4)	218.8 (± 830.2)	0.003
Thrombocytopenia (< 150 × 10˄9/L), *n* (%)	26 (± 39)	35 (± 18)	0.002
Neutropenia (< 500 × 10˄9/L), *n* (%)	9 (± 14)	1 (± 1)	0.003
Creatinin, mg/dl	1.1 (± 1.3)	1.4 (± 2.3)	0.318
Albumin, g/dl	35.6 (± 6.7)	37.4 (± 5.5)	0.003
D-dimer, µg/L	1229.2 (± 1200.3)	947.7 (± 994)	0.093
C-Reactive Protein, mg/dL	6.574 (± 8.417)	7.062 (± 7.522)	0.650
Ferritin, µg/L	1472.2 (± 1903.3)	752.3 (± 794.1)	0.008
LDH, U/L	341.4 (± 160.3)	311.5 (± 133.1)	0.174
Procalcitonin, ng/ml	0.3 (± 0.5)	0.9 (± 3)	0.117
Features at the respiratory worsening**			
Worsening during hospitalization, *n* (%)	52 (78.7)	129 (65.5)	0.04
FiO_2_ mean (SD)	39.3 (± 22.1)	59.8 (± 23.7)	< 0.001
PO_2/_FiO_2,_ mean (SD)	259.2 (± 115.8)	208.7 (± 82.7)	0.003
Days from symptoms onset to worsening, mean (SD)	13.20 (± 11.69)	9.45 (± 5.38)	0.041
Days from SARS-CoV2 diagnosis to worsening, mean (SD)	11.63 (± 13.69)	5.69 (± 4.4)	0.006
Days from admission to worsening, mean (SD)	12.3 (± 12.3)	3.3 (± 3.1)	0.001
Days from thrombocytopenia to worsening, mean (SD)	5.7 (± 10.4)	3.0 (± 2.3)	0.119
Days from neutropenia to worsening, mean (SD)	1.2 (± 5.2)	1.2 (± 5.5)	0.897
Radiological features, mean (SD)			
Percentage of total lung parenchyma involvement	39 (26.3)	NA	–
Total CT score	6 (6.2)	NA	–
Percentage of lung parenchyma involvement at CT1	22.58 (22.19)	NA	–
CT score at CT1	5.49 (5.55)	NA	–
Percentage of lung parenchyma involvement at CT2	36.31 (24.55)	NA	–
CT score at CT2	8.71 (6.02)	NA	–
Therapy, *n* (%)			
Remdesivir	31 (47)	79 (40)	0.250
Enoxaparin	48 (73)	161 (81)	0.234
Corticosteroids	60 (91)	145 (73)	< 0.001
Days of corticosteroids	18.1 (22.9)	8.1 (4.3)	< 0.001
Baricitinib	1 (2)	0 (0)	0.321
Convalescent plasma	8 (12)	0 (0)	0.004
Antibiotics	55 (83)	128 (65)	< 0.001
Outcomes			
Intensive Care Unit admission, *n* (%)	18 (27)	16 (8)	0.002
In-hospital mortality, *n* (%)	23 (35)	20 (10)	< 0.001
Days of viral shedding, mean (SD)	35.4 (± 23.4)	19.4 (± 10.2)	0.010
Secondary infections, *n* (%)	26 (39)	58 (29)	0.137
MDRO colonization	1 (2)	2 (1)	0.912
Opportunistic infections	8 (12)	0 (0)	0.004

*other include headache, diarrhea and pharyngodynia; **respiratory worsening was defined as: i) the need of supplementary oxygen therapy or ii) the need of increasing oxygen therapy supplementation in a patient with SARS-CoV2 infection for reasons directly related to the infection. A careful evaluation of causes of supplementary oxygen therapy for reasons other than SARS-CoV2 infection (i.e. cardiac failure, bacterial superinfections) was performed. In the case of doubt, a panel discussion was performed. *MDRO* Multidrug resistant organisms.

Amongst cases, non-Hodgkin lymphoma (NHL) was observed in 27 (41%) of the subjects, followed by multiple myeloma (9, 14%), acute myeloid leukemia (7, 11%), chronic lymphocytic leukemia (7, 11%) and acute lymphocytic leukemia (5, 8%). Fifteen subjects (23%) have received a new diagnosis of haematological malignancy in the last months and, therefore, have not yet started or concluded treatment for the underlying disease, 34 (52%) were in complete or partial remission, 12 (18%) were suffering a relapsing or refractory disease, while 4 (6.1%) still had a not defined disease status. Prior (90 d) active treatment was given in 36 subjects (54.5%) [chemotherapy, *n* = 17, immunotherapy, *n* = 9, chemotherapy + immunotherapy, *n* = 10] and, amongst them, 12 (18%) had received recent (30 d) chemotherapy. Only four patients (6%) had a history of allogeneic stem cell transplantation (Supplementary Table 1).

Concerning the treatment of SARS-CoV2 infection, no differences emerged regarding the use of remdesivir and enoxaparin between cases and controls (47% vs 40%, *p* = 0.250 and 73% vs 81%, *p* = 0.234, respectively). In contrast, corticosteroid and antimicrobial therapies were significantly more frequent in cases than in controls (91% vs 73%, *p* < 0.001 and 83% vs 65%, *p* < 0.001, respectively). Of note, also the duration of corticosteroids treatment was longer in patients with haematological malignancies (18.1 ± 22.9 vs 8.1 ± 4.3 days, *p* < 0.001).

**Fig. 1:**
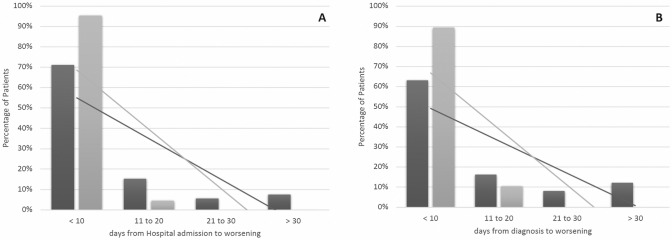
Differences in the respiratory worsening between cases (black column) and controls (grey column). Panel **A** Differences in the days from hospital admission to respiratory worsening. Panel **B** Differences in the days from SARS-CoV-2 infection diagnosis to respiratory worsening

**Fig. 2:**
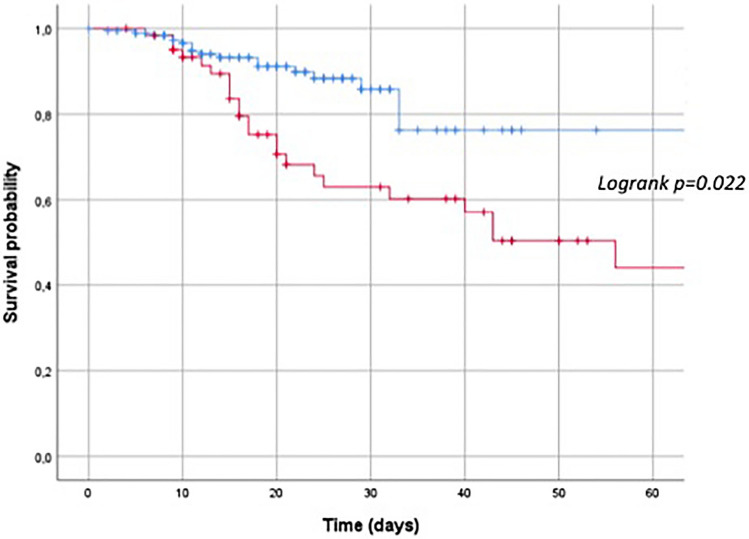
Cumulative proportion of mortality rate between patients with haematological malignancies. Cases: red line; controls: blue line

Super-infection rate and colonization by multi-drug resistant pathogens were similar in the two groups, whereas, as expected, opportunistic infections more frequently affected the haematological ones (*p* = 0.004). Furthermore, cases had a significantly longer duration of hospitalization (31.79 ± 28.04 vs 19.16 ± 13.39 days, *p* = 0.001) as well as a longer duration of viral shedding (35.4 ± 23.4 vs 19.4 ± 10.2 days, *p* = 0.010) than controls. Compared to controls, cases experienced higher rates of respiratory deterioration (78.7% vs 65.5%, *p* = 0.04). Furthermore, the time from symptoms onset, hospital admission and SARS-CoV2 diagnosis to respiratory worsening was significantly longer in cases [13.20 ± 11.69 vs 9.45 ± 5.38 days (*p* = 0.041), 12.3 ± 12.3 vs 3.3 ± 3.1 days (*p* = 0.001) and 11.63 ± 13.69 vs 5.69 ± 4.39 days (*p* = 0.006), respectively], leading to a delayed respiratory deterioration in cases compared to controls. As shown in Fig. 1, 29% of cases developed respiratory worsening more than 10 days after hospital admission, compared to 5% in the control group (Fig. 1, Panel A); likewise, 37% of cases worsened more than 10 days after SARS-CoV2 diagnosis, compared to 11% of controls (Fig. 1, Panel B). Cases had higher rates of ICU admission and in-hospital mortality as compared to controls: 27% vs 8%, (*p* = 0.002) and 35% vs 10%, (*p* < 0.001), respectively (Fig. 2).

At multivariable analysis, having an haematological malignancy (i.e. belonging to the case group) [OR 4.76 (2.4–9.4), *p* < 0.001], thrombocytopenia at admission [OR 3.03 (1.6–5.6), *p* < 0.001], neutropenia at admission [OR 31.1 (3.86–250.7), *p* = 0.001], reduced level of serum albumin [OR 3.1 (2.1–6.6), *p* = 0.001], chronic corticosteroid use [OR 3.65 (1.49–8.96), *p* = 0.004], prior infection [OR 57.7 (3.2–1014.4), *p* = 0.006], low PaO2/FiO2 ratio at the time of respiratory worsening [OR 2.2 (1.3–10.4), *p* = 0.022] and a longer time to respiratory deterioration [OR 3.3 (2–12.4), *p* = 0.002] were independent risk factors for in-hospital mortality (Table 2).

**Table 2:** Multivariable analysis on independent risk factors for in-hospital mortality in the whole study population

	ORs (95%CI)	*P value*
Haematological malignancy	4.76 (2.4–9.4)	< 0.001
Thrombocytopenia at hospital admission^1^	3.03 (1.6–5.6)	< 0.001
Neutropenia at hospital admission^2^	31.1 (3.86–250.7)	0.001
Low albumin at hospital admission^3^	3.1 (2.1–6.6)	0.001
Days from admission to respiratory worsening* (> 10d)	3.3 (2–12.4)	0.002
Corticosteroids treatment	3.65 (1.49–8.96)	0.004
Prior (30-d) infection	57.7 (3.2–1014.4)	0.006
PO_2/_FiO_2_ at respiratory worsening* (< 250)	2.2 (1.3–10.4)	0.022

^*1*^Thrombocytopenia was defined as platelets count < 150 × 10˄9/L; ^2^Neutropenia was defined as polymorphonuclear leukocytes count < 500 × 10˄9/L; ^3^level of serum albumin < 3.5 g/dl. *respiratory worsening was defined as: i) the need of supplementary oxygen therapy or ii) the need of increasing oxygen therapy supplementation in a patient with SARS-CoV2 infection for reasons directly related to the infection. A careful evaluation of causes of supplementary oxygen therapy for reasons other than SARS-CoV2 infection (i.e. cardiac failure, bacterial superinfections) was performed. In the case of doubt, a panel discussion was performed

As shown in Table 3, independent risk factors for mortality in patients with haematological malignancies were neutropenia at hospital admission [OR 3.1 (2.9–270), *p* < 0.001], prior infection [OR 7.7 (3.2–112), *p* < 0.001], a longer time to respiratory deterioration [OR 4.1 (2.2–12.4), *p* < 0.001], active treatment [OR 4.4 (1.5–22.3), *p* < 0.001], multiple myeloma [OR 1.5 (1.1–3.3), *p* = 0.044], the variation of the total lung parenchyma involvement (CT1-CT2) [OR 2.6 (1.4–4.8), *p* = 0.004] and the variation of the CT lung score during hospitalization [OR 2.6 (1.2–12.2), *p* = 0.006].

**Table 3:** Analysis of risk factors for in-hospital mortality in patients with haematological malignancies

	ORs (CIs 95%)	*P value*
Thrombocytopenia at hospital admission^1^	1.2 (0.8–7.6)	0.212
Neutropenia at hospital admission^2^	3.1 (2.9–270)	< 0.001
Low albumin at hospital admission^3^	1.4 (0.9–6.6)	0.086
Days from admission to respiratory worsening*(> 10d)	4.1 (2.2–12.4)	< 0.001
Corticosteroids	1.7 (0.88–9.6)	0.154
Prior (30 d) infections^6^	7.7 (3.2–112)	< 0.001
PO_2/_FiO_2_ at respiratory worsening* (< 250)	1.2 (0.7–7.2)	0.082
Days from SARS-CoV-2 diagnosis to respiratory worsening (> 10-d)	2.8 (1.4–22.1)	0.014
Percentage of total lung parenchyma involvement variation (CT1-CT2)	2.6 (1.4–4.8)	0.004
Total CT score variation (CT1-CT2)	2.4 (1.2–12.2)	0.006
Active treatment in the last 90 days	4.4 (1.5–22.3)	< 0.001
Multiple myeloma	1.5 (1.1–3.3)	0.044

^*1*^Thrombocytopenia was defined as platelets count < 150 × 10˄9/L; ^2^Neutropenia was defined as polymorphonuclear leukocytes count < 500 × 10˄9/L; ^3^level of serum albumin < 3.5 g/dl. *respiratory worsening was defined as: i) the need of supplementary oxygen therapy or ii) the need of increasing oxygen therapy supplementation in a patient with SARS-CoV2 infection for reasons directly related to the infection. A careful evaluation of causes of supplementary oxygen therapy for reasons other than SARS-CoV2 infection (i.e. cardiac failure, bacterial superinfections) was performed. In the case of doubt, a panel discussion was performed

In patients under active treatment, those receiving only immunotherapy (n = 9) had a mortality rate of 11.2% (1/9), compared to 29.4% (5/17) and 30% (3/10) of chemotherapy only and chemotherapy + immunotherapy, respectively.

Active treatment [OR 3.2 (1.8–12.3), *p* < 0.001], the variation of the CT lung score during hospitalization [OR 1.4 (1.1–8.2), *p* < 0.001], the variation of the total lung parenchyma involvement (CT1-CT2) [OR 1.6 (1.2–6.8), *p* < 0.001] and non-Hodgkin lymphoma [OR 1.4 (1.1–10.3), *p* = 0.011] were associated with in-hospital development of respiratory deterioration in cases (Supplementary Table2).

## Discussion

Our data support previous observations that, amongst patients with COVID-19, those with haematological malignancies have higher mortality and ICU admission rates than subjects without malignancies [3, 11, 21–24]. Furthermore, they have increased risk in the presence of neutropenia at hospital admission, prior infection, active chemotherapy treatment and multiple myeloma [4, 8].

Nevertheless, in our opinion, the most interesting observation of this report was that i) haematological patients experienced a higher rate of respiratory deterioration than controls, ii) that this complication occurred later during the disease and, notably, iii) that the longer time to respiratory deterioration together with the increase of the CT lung score during hospitalization represented independent risk factors for mortality.

To point out the different clinical course between the two groups, we analysed the PaO2/FiO2 ratio at the time of worsening and we found that haematological patients experienced a higher rate of worsening than controls, although the PaO2/FiO2 ratio at the time of deterioration was still higher than in subjects without malignancies, apparently in contrast with the worse observed outcome. Furthermore, deterioration occurred more than 10 days after hospital admission in 29% of cases and only in 5% of controls. Being a novel aspect of the infection’s clinical course, the finding that haematological patients experienced a delayed respiratory worsening still has no clear explanation. Indeed, on one hand the immunocompromised status may lead the virus to replicate and to persist for a longer time; on the other hand, the immune response to the viral infection is delayed [12, 22, 25]. It may also be speculated that, as far as the restoration of the immune system occurs, for instance after chemotherapy-induced amelioration of the haematological disease, patients experience an excessive inflammatory response, which, eventually, is responsible for the clinical observed deterioration.

Another interesting finding of the present study was the correlation between the respiratory worsening and the radiology pattern in patients with haematological malignancies [26]. Although CT severity score has been shown to be a predictor of death in patients with COVID-19 pneumonia [27, 28], very few data are present regarding the role of this radiological parameter in patients with haematological malignancies. In this special context, we showed that not the CT score itself but its change during hospitalization was predictive of worse outcomes in haematological patients and, therefore, it may be used as a feature of clinical worsening.

Corticosteroids use and duration of treatment were significantly higher in cases than in controls, suggesting that the immune system of this special population is not only affected by the malignancy itself but also by its treatment. Likewise, cases had a higher rate of hypoalbuminemia at hospital admission than controls, confirming its prognostic role for mortality, prolonged viral shedding and coagulopathy during COVID-19 pandemic [18, 29–31] as well as for morbidity and mortality in several malignancies, including the haematological ones [32, 33].

Patients with haematological malignancies often experience prolonged shedding of viral RNA from the upper respiratory tract. In line with this, we showed that duration of viral positivity was significantly longer in patients with haematological malignancies than those without. With the continuous emergence of new variants, this phenomenon may be of significant concern, since it seems to contribute to the selection of sequence SARS-CoV-2 variants within the same host [34–36].

Our study has several limitations. First, its retrospective and single-centre nature. Second, we could not establish with certainty whether the diagnosis of SARS-CoV2 infection would delay the treatment of the haematological malignancy. This leads to a possible additional reason explaining the observed high mortality. Furthermore, groups were not perfectly comparable with regard to therapy; for instance, corticosteroids were used more in cases than in controls because of the haematological disease, even if not indicated for the treatment of COVID-19. Nevertheless, to reduce the possible bias of subjects’ selection, such as the different pandemic waves, we performed a strict match for age, sex and period of hospitalization. The level of the inflammatory marker IL-6 was not systematically measured at hospital admission in all the patients and, therefore, we could not evaluate whether it would have an influence on the observed different clinical course of infection between cases and controls. The low absolute number of haematological patients receiving only immunotherapy did not allow us to perform subgroup analyses for the outcome. At last, we enrolled patients before the onset of SARS-CoV-2 vaccination and/or the introduction of monoclonal antibodies in the clinical practice and therefore our findings could not lead to a generalization of the results. Notwithstanding, we were able to describe the singular clinical course of the infection in this special population and our results may represent the starting point for additional studies evaluating if, and how, the course of the disease has changed after the introduction of vaccination or monoclonal antibodies.

In conclusion, an underlying haematological malignancy was associated with a worse clinical outcome in our COVID-19 patients. Furthermore, haematological patients experienced a peculiar clinical course of the infection. This consisted in a higher rate of worsening and, above all, in a longer time to respiratory deterioration compared to non-haematological ones, which independently predicts mortality. Therefore, a careful and prolonged clinical monitoring is needed in this special population.

## Supplementary Information

Below is the link to the electronic supplementary material.

Supplementary file1 (DOCX 15 KB)

Supplementary file2 (JPG 47 KB)

Supplementary file3 (DOCX 16 KB)

## Data Availability

All data relevant to the study are included in the article or uploaded as supplementary information and are available from the corresponding author upon request.
