# Supplementary material for: Clinical course of Coronavirus Disease-19 in patients with haematological malignancies is characterized by a longer time to respiratory deterioration compared to non-haematological ones: results from a case–control study
Source: Infection. 2022 Jul 3;50(5):1373–82. doi: 10.1007/s15010-022-01869-w (PMC9251021; doi:10.1007/s15010-022-01869-w)
Supplement: Supplementary file 1 — Supplementary file1 (DOCX 15 KB) [file 15010_2022_1869_MOESM1_ESM.docx]

|  | ORs (CIs 95%) | *p-value* |
| --- | --- | --- |
| Thrombocytopenia at hospital admission^1^ | 1.9 (0.5-7.8) | 0.212 |
| Neutropenia at hospital admission^2^ | 1.2 (0.1-22.2) | 0.888 |
| Low albumin at hospital admission^3^ | 1.0 (0.9-1.1) | 0.087 |
| Corticosteroids | 1.7 (0.88-9.6) | 0.154 |
| Prior (30-d) infections | 0.8 (0.1-5.2) | 0.191 |
| Percentage of total lung parenchyma involvement variation (CT1-CT2) | 1.6 (1.2-6.8) | <0.001 |
| Total CT score variation (CT1-CT2) | 1.4 (1.1-8.2) | <0.001 |
| Active treatment in the last 90 days | 3.2 (1.8-12.3) | <0.001 |
| Non Hogkin Lymphoma | 1.4 (1.1-10.3) | 0.011 |

*^1^:*Thrombocytopenia was defined as platelets count <150 x 10˄9/L; *^2^:*Neutropenia was defined as polymorphonuclear leukocytes count <500 x 10˄9/L; *^3^:*level of serum albumin < 3.5 g/dl.

**Supplementary Table2.** Multivariable analysis of risk factors for respiratory worsening in patients with haematological malignancies. Respiratory worsening was defined as: i) the need of supplementary oxygen therapy or ii) the need of increasing oxygen therapy supplementation in a patient with SARS-CoV2 infection for reasons directly related to the infection. A careful evaluation of causes of supplementary oxygen therapy for reasons other than SARS-CoV2 infection (i.e. cardiac failure, bacterial superinfections) was performed. In the case of doubt, a panel discussion was performed.
