# Supplementary figures and images for: Clinical course of Coronavirus Disease-19 in patients with haematological malignancies is characterized by a longer time to respiratory deterioration compared to non-haematological ones: results from a case–control study
Source: Infection. 2022 Jul 3;50(5):1373–82. doi: 10.1007/s15010-022-01869-w (PMC9251021; doi:10.1007/s15010-022-01869-w)

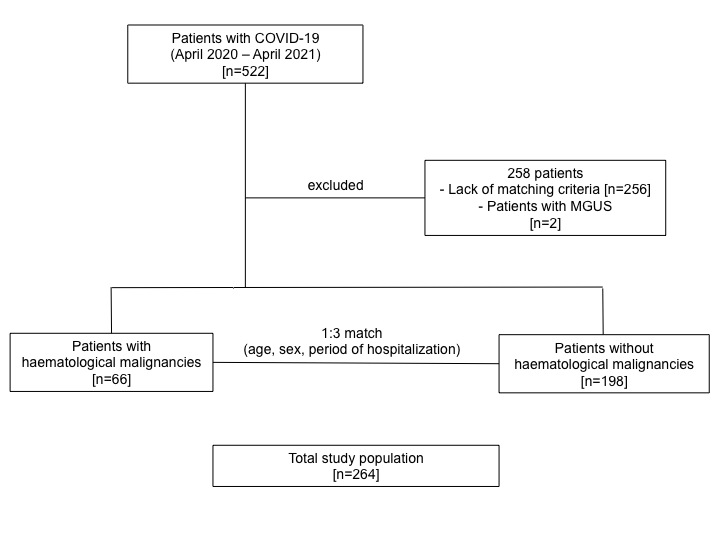

Supplement: Supplementary file 2 — Supplementary file2 (JPG 47 KB) [file 15010_2022_1869_MOESM2_ESM.jpg]
