# Supplementary material for: Clinical course of Coronavirus Disease-19 in patients with haematological malignancies is characterized by a longer time to respiratory deterioration compared to non-haematological ones: results from a case–control study
Source: Infection. 2022 Jul 3;50(5):1373–82. doi: 10.1007/s15010-022-01869-w (PMC9251021; doi:10.1007/s15010-022-01869-w)
Supplement: Supplementary file 3 — Supplementary file3 (DOCX 16 KB) [file 15010_2022_1869_MOESM3_ESM.docx]

| **Type of haematological disease** | **N (%)** |
| --- | --- |
| Non-Hodgkin lymphoma | 27 (41) |
| Multiple myeloma | 9 (14) |
| Acute myeloid leukemia | 7 (11) |
| Chronic lymphocytic leukemia | 7 (11) |
| Acute lymphocytic leukemia | 5 (8) |
| Myelofibrosis | 3 (5) |
| Chronic myeloid leukemia | 0 (0) |
| Pro-lymphocytic leukemia | 1 (2) |
| Plasma cell leukemia | 1 (2) |
| Essential thrombocythemia | 1 (2) |
| Myelodysplastic syndrome | 1 (2) |
| Hodgkin lymphoma | 1 (2) |
| Polycythemia vera | 1 (2) |
| Langerhans cell histiocytosis | 1 (2) |
| **Disease status at the diagnosis of SARS-CoV2 infection°** |  |
| New diagnosis | 15 (23) |
| Complete or partial remission | 34 (52) |
| Relapsing or refractory disease | 12 (18) |
| Not defined disease status | 4 (6) |
| **Treatment of the underlying malignancy** |  |
| Active treatment in the last 90 days* | 36 (54.5) |
| Chemotherapy | 17 (26) |
| Immunotherapy** | 9 (14) |
| Chemotherapy plus immunotherapy | 10 (15) |
| Chemotherapy in the last 30 days | 12 (18) |
| Prior/concluded treatment | 22 (33) |
| No treatment | 10 (15) |
| Allogeneic stem cell transplantation | 4 (6) |

°: Status of haematological malignancy was defined as new diagnosis, refractory or relapsing disease, according to the guidelines of European Society for Medical Oncology [16]. *: Prior active treatment included the receipt of chemotherapy or immunotherapy, or both, in the previous 90 days. Immunotherapy included the receipt of monoclonal antibodies as rituximab, daratumumab, obinutuzumab and tyrosine kinase inhibitors as imitinib, ibrutinib, ruxolitinib, venetoclax. **: rituximab (n=3); inotuzumab (n=1); Obinutuzumab (n=1); ibrutinib (n=1); ruxolitinib (n=2); imatinib (n=1).

**Supplementary Table 1.** Disease features of patients with haematological malignancy.
